# Ultrafast plasma method allows rapid immobilization of monatomic copper on carboxyl-deficient g-C_3_N_4_ for efficient photocatalytic hydrogen production

**DOI:** 10.3389/fchem.2022.972496

**Published:** 2022-08-26

**Authors:** Shuchang Xu, Zhihao Zhang, Daqian Wang, Junyang Lu, Ying Guo, Shifei Kang, Xijiang Chang

**Affiliations:** ^1^ College of Science, Donghua University, Shanghai, China; ^2^ Department of Environmental Science and Engineering, University of Shanghai for Science and Technology, Shanghai, China; ^3^ Shanghai Yiming Filtration Technology Co., Ltd., Shanghai, China; ^4^ Magnetic Confinement Fusion Research Center of Ministry Education, Donghua University, Shanghai, China; ^5^ Institute of Photochemistry and Photocatalyst, University of Shanghai for Science and Technology, Shanghai, China

**Keywords:** Cu-g-C_3_N_4_, single-atom catalyst (SAC), carboxyl defect, plasma method, reactive metal-support interactions

## Abstract

Transition-metal monometallic photocatalysts have received extensive attention owing to the maximization of atomic utilization efficiency. However, in previous related works, single-atom loading and stability are generally low due to limited anchor sites and mechanisms. Recently, adding transition-metal monatomic sites to defective carbon nitrides has a good prospect, but there is still lack of diversity in defect structures and preparation techniques. Here, a strategy for preparing defect-type carbon-nitride–coupled monatomic copper catalysts by an ultrafast plasma method is reported. In this method, oxalic acid and commercial copper salt are used as a carboxyl defect additive and a copper source, respectively. Carbon nitride samples containing carboxyl defects and monatomic copper can be processed within 10 min by one-step argon plasma treatment. Infrared spectroscopy and nuclear magnetic resonance prove the existence of carboxyl defects. Spherical aberration electron microscopy and synchrotron radiation analysis confirm the existence of monatomic copper. The proportion of monatomic copper is relatively high, and the purity is high and very uniform. The Cu PCN as-prepared shows not only high photo-Fenton pollutant degradation ability but also high photocatalytic hydrogen evolution ability under visible light. In the photocatalytic reaction, the reversible change of Cu^+^/Cu^2+^ greatly promotes the separation and transmission of photogenerated carriers and improves the utilization of photoelectrons. The photocatalytic hydrogen evolution rate of the optimized sample is 8.34 mmol g^−1^·h^−1^, which is 4.54 times that of the raw carbon nitride photocatalyst. The cyclic photo-Fenton experiment confirms the catalyst has excellent repeatability in a strong oxidation environment. The synergistic mechanism of the photocatalyst obtained by this plasma is the coordination of single-atom copper sites and carboxyl defect sites. The single copper atoms incorporated can act as an electron-rich active center, enhancing the h+ adsorption and reduction capacity of Cu PCN. At the same time, the carboxyl defect sites can form hydrogen bonds to stabilize the production of hydrogen atoms and subsequently convert them to hydrogen because of the unstable hydrogen bond structure. This plasma strategy is green, convenient, environment-friendly, and waste-free. More importantly, it has the potential for large-scale production, which brings a new way for the general preparation of high-quality monatomic catalysts.

## Introduction

The accelerated growth of human activities and excessive combustion of fossil fuels have led to a steady increase in carbon dioxide levels, threatening the sustainable development of the society and economy. Solar energy decomposition of water to produce hydrogen is a promising strategy for future energy conversion. Although the existing basic photocatalysts such as TiO_2_ and carbon nitride (g-C_3_N_4_) are widely used owing to their low costs and high stability. They have some shortcomings, such as the fast recombination rate of photogenerated carriers and slow proton reduction kinetics. Therefore, these problems shall be solved in order to strengthen charge separation and H_2_ synthesis. Doping, defect engineering, heterojunction, morphology control, and other strategies are often adopted to reduce photogenerated carrier recombination and improve H_2_ precipitation. Among them, loading cocatalysts on the surface of basic photocatalysts and establishing metal-semiconductor Schottky junctions are an effective way to enhance charge separation. Schottky junctions not only facilitate the extraction of photogenerated electrons but also significantly reduce the energy barrier of proton reduction. Pt, Au, Pd, and other noble metals are widely used as photocatalysts because of their low activation energy and high charge separation ability. For example, Pt loading improves the hydrogen production performance of TiO_2_ by 129 times. However, precious metal elements have a high cost and metal particles agglomeration effect and do not reach the ideal actual effect, which greatly hinders their commercial applications to sustainable energy development (He et al., 2022).

To fundamentally reduce the cost of noble metal loading, build an ideal metal-semiconductor Schottky junction photocatalysis platform, and truly improve practicality, researchers need a strategy to design and prepare transition-metal single-atom catalysts (SACs) supported on basic photocatalysts. In recent years, much research has been carried out on transition metals (e.g., Cu, Ni, Co, and Fe) with rich reserves to replace these precious metals for photocatalysis cocatalysts. Moreover, because the atom utilization rate is low when the particle size of transition-metal cocatalysts is large, no breakthrough can be made in improving the efficiency of photocatalysts loaded with a cocatalyst. Recently, monatomic catalysts have attracted much attention owing to their ideal maximization of reaction active sites (Wang B. et al., 2019). The isolated metal atoms immobilized on the photocatalyst provide more adsorption sites and active reaction sites for water molecules and have excellent reaction kinetic properties similar to homogeneous catalysis. So far, there are a few reports on the photocatalytic hydrogen evolution and pollutant degradation of supported transition-metal monatomic catalysts. However, in the catalytic reaction, aggregation of single atoms is inevitable due to the high surface energy of single atoms or unstable anchoring because most single atoms are synthesized by post-treatment processes (e.g., impregnation). Generally, a larger percentage of single atoms leads to higher activity and worse stability. Therefore, obtaining highly dispersed and concentrated monatomic catalysts is still the main bottleneck of photocatalytic hydrogen production (Zhang et al., 2022).

Single-metal-atom photocatalysts have received extensive attention given the rational use of metal resources and the maximum atomic utilization efficiency. However, the loading of single atoms in previous related work is generally low, which is because the proportion of binding sites suitable for the existence and anchoring of single atoms in conventional photocatalysts is very low. N-rich g-C_3_N_4_ has a large vacancy structure and electronegative n sites owing to its unique heptazine ring structure. The carrier-containing nitrogen vacancy can provide three sp2 hybrid N atoms to anchor different metal oxides and single atoms. Thus, N-rich g-C_3_N_4_ is used as a carrier to fix single transition-metal atoms. However, due to excessive defects in the overall morphology and amorphous g-C_3_N_4_, the enhancement in the photocatalytic activity of g-C_3_N_4_ by introducing single atoms is limited. In addition, the long-term stability of single atoms is still a problem if no reasonable stability mechanism can be provided (Li et al., 2018).

In this view, the academic community has recently reached a consensus that adding transition-metal monatomic sites to defective carbon nitride has a good prospect. Studies on electrocatalysis show that the transition-metal atoms supported on g-C_3_N_4_ with N vacancies are stable and the strong reactive metal-support interactions between transition-metal atoms and the N-containing vacancy graphite carbonitride ensure the stability of the composite system (Niu et al., 2021). Density functional theory (DFT) analysis shows that most isolated metal atoms (Ti, V, Co, Ni, Zr, Mo, Ru, and Pt) can be immobilized on nitrogen vacancies and remain stable after molecular dynamics structural relaxation. These results show that based on the defect engineering control of g-C_3_N_4_, the active sites and catalytic performance can be effectively improved by using the nitrogen vacancy of the catalyst to anchor the single transition-metal atoms (Chen et al., 2018).

At present, the strategy of defect engineering combined with the construction of monatomic catalysts is rarely reported in the field of photocatalysis. More importantly, the defect types and treatment technologies are relatively limited. In terms of defect types, most reports focus on N defects (Wu et al., 2022). However, the nonspecificity of N defects makes it difficult to understand the mechanism of synergistic catalysis and stabilization. In addition, more available defect configurations shall be selected, such as carboxyl defect configurations that are proved effective in regulating electronic structure reconstruction and promoting carrier separation (Yang et al., 2021). Furthermore, the preparation methods are mostly limited to the chemical method and microwave method, indicating more new technologies and strategies with broader possibilities are needed to promote the development and industrial application of the technical route of defect-supported fixed monatomic catalysts.

Given the technology of preparing monatomic catalysts and producing surface carboxyl groups, the emerging cold plasma technology seems promising (Li et al., 2021). The atmospheric pressure dielectric barrier discharges act as promising alternative to other surface treatments, such as diazonium and alkali hydrolytic treatments, for carboxyl functionalization of polylactic acid (Durán et al., 2020). In particular, the microwave cold plasma used here has incomparable advantages and characteristics compared with other plasma methods. Microwave plasma has the advantages of no electrode, large volume, wide operating pressure, low energy consumption, high efficiency, and low cost. However, so far, there are few reports on the modification of microwave plasma equipment and materials for simultaneous regulation of monatomic and carboxyl groups. Precise plasma surface modification and catalyst energization for carbon nitride polymer semiconductors are highly needed.

In this view, based on the previous work on controlling the defect structure of carbon nitride photocatalysts by plasma technology (Kang et al., 2020; Kang et al., 2019a; Kang et al., 2019b), we developed a general synthesis strategy using oxalic acid as the carboxyl defect promoter to immobilize monatomic Cu catalysts onto carboxyl defect g-C_3_N_4_ by an ultrafast plasma method. The single Cu atom/crystal g-C_3_N_4_ photocatalyst (Cu-CN-COOH) as-prepared showed effective photocatalytic hydrogen evolution without any cocatalyst or sacrificial agent. This synthesis strategy has very strong universality and is expected to effectively fix various monatomic catalysts (Pt, Cu, and Ni) on different supports. At the same time, the monatomic catalyst prepared by the general monatomic catalyst loading method has a similar chemical environment and thus can be used as an ideal model to compare the intrinsic activity differences among monatomic metal species.

## Experimental

### Material preparation and the synthesis of raw g-C_3_N_4_

All chemicals were provided by Adamas Reagents and used as received. The untreated raw g-C_3_N_4_ was prepared by directly heating urea from 20 to 550°C in a covered ceramic crucible. The heating rate was 5°C min^−1^. Then, the light-yellow solid powder obtained was used as the raw material and platform to study the potential of the plasma method in regulating Cu-SAC in defective g-C_3_N_4_ photocatalysts. CuSO_4_ and oxalate were used as the Cu-SAC and -COOH precursors, respectively.

### Setup of the plasma equipment and plasma treatment process

**FIGURE 1:**
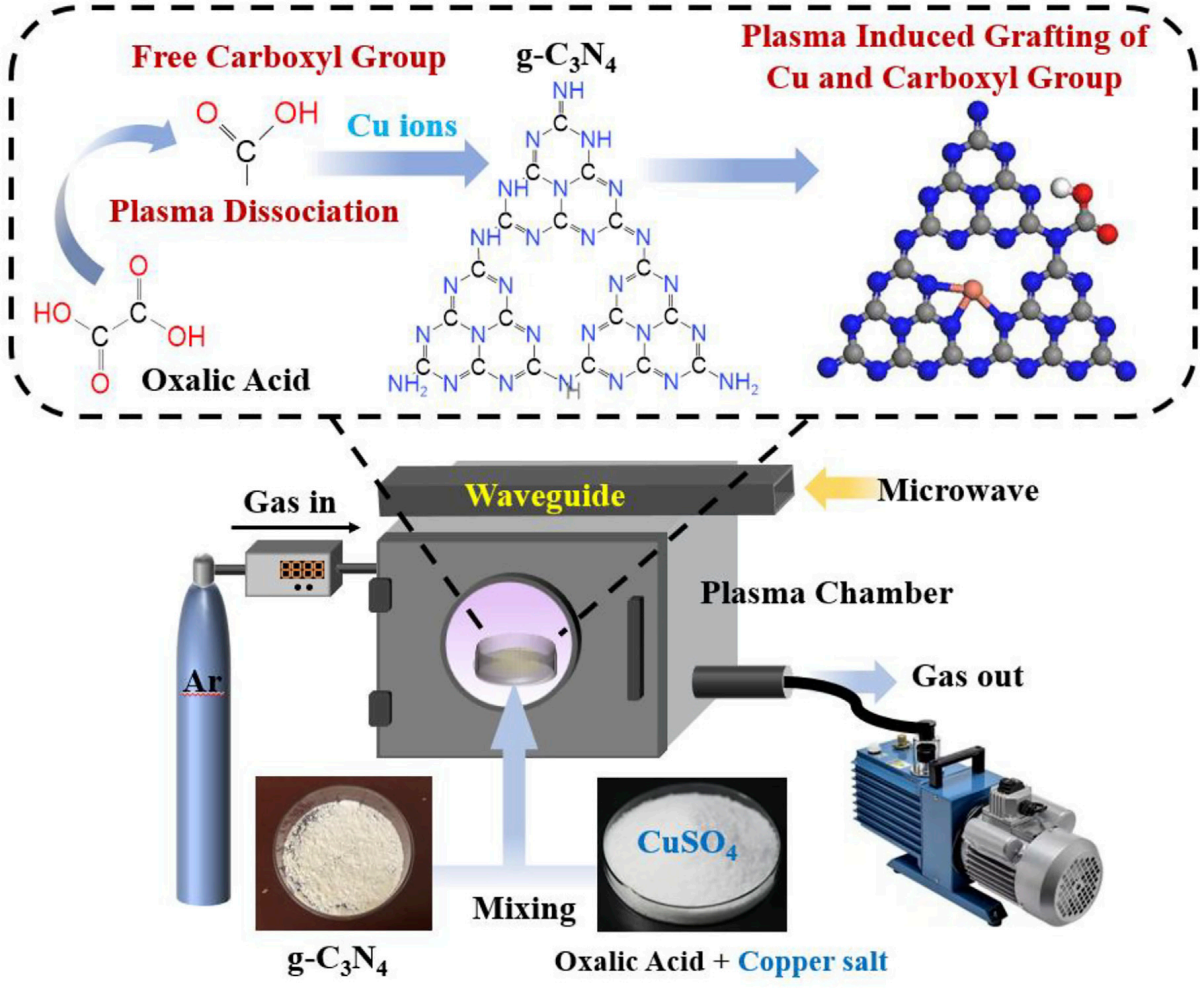
Schematic diagram of the plasma equipment used in this work and the molecular processes.

Figure 1 shows the setup of the plasma equipment used in this work and the proposed molecular processes during the treatment. The Cu species and -COOH defect endowed sample was obtained by direct plasma immersion of the mixture of raw g-C_3_N_4_, oxalate (1 wt%), and CuSO_4_ (1 wt%), and the obtained sample was named as Cu-pCN-oxalate. The Cu-pCN and pCN-oxalate controls were obtained by the same system without the presence of oxalate and CuSO_4_, respectively. In determining the suitable proportion of copper salt, we mainly refer to the metal content of most conventional SAC Cu catalysts. If the copper content is too high, the purity or stability of the monatomic copper system will be significantly reduced. According to DFT calculation and considering the volatilization of oxalic acid, we believe that the same mass fraction of oxalic acid is reasonable.

The high-activity and high-energy environment of microwave surface wave plasma discharge provides a greater possibility for catalyst surface modification. At the same time, as a nonthermal plasma treatment, it does not affect the main molecular structure and photocatalytic band structure of the polymer semiconductor substrate. As a mature plasma process, although the microwave surface wave plasma technique is not widely used because of its relatively high cost, this technology has the following irreplaceable advantages when compared with the conventional dielectric barrier discharge plasma methods. 1) It has high degrees of ionization and decomposition. 2) The ratios of electron temperature and ion temperature to neutral gas temperature are very high, and the carrier gas maintains an appropriate temperature. This feature, in the case of vapor deposition, can cause the substrate to not be at too high temperature. 3) It can maintain plasma at high pressure. 4) There is no internal electrode and no substance other than working gas in the plasma container, which is clean and free of pollution sources. The plasma generator can maintain long life. 5) The plasma from magnetic constraint can be adopted, which is constrained in the agreed space, and the microwave junction and magnetic circuit can be compatible. 6) The safety factor is high. The high voltage source and the plasma generator are isolated from each other, which cannot be achieved by dielectric barrier discharge plasma. The microwave leakage is insignificant and easily reaches the radiation safety standard, which can be hardly achieved by high-frequency induction plasma. 7) The microwave generator is stable and easy to control. 8) Microwave plasma is a relatively quiet process in many cases, which avoids the high noise faced by dielectric barrier discharge (Crawford, 1971; Ohtsuki and Ofuruton, 1991; Kelly and Bogaerts, 2021).

Considering the high difficulty to modify the carboxyl group through the gas source (carboxyl-related plasma species are extremely unstable with short transient life, and universal treatment with the scale effect is hard to achieve), we creatively mixed a certain proportion of oxalic acid into the polymer semiconductor samples, which were treated as the main source of the carboxyl group. The molecular structure of oxalic acid was split into halves, which are two high-quality and highly active carboxyl precursor ions. This carboxyl source is milder and more effective than direct forcing oxidation of carbon sites in carbon nitride. Given that the preparation of monatomic catalysts by plasma requires stable oxidation conditions (excessive oxidation will lead to a collapse of the metal coordination structure and the agglomeration of metal particles), this unique strategy of introducing a carboxyl precursor chemical reagent into the material is very suitable for monatomic modification and carboxyl defect structure regulation at the same time. The nontoxic and noncorrosive oxalic acid is an inexpensive chemical and food industrial raw material, which makes the cold plasma technology and overall strategy in this study feasible for industrial scale-up.

### Catalyst characterization

X-ray diffraction (XRD) analyses were performed on a Bruker D8 Advance diffraction meter. Fourier transform infrared (FT-IR) spectra were collected by a Thermo Scientific Nicolet iS50 FT-IR spectrometer. UV-Vis diffuse reflectance spectra (DRS) were recorded by using a Shimadzu UV-2600 spectrometer. The porous features were measured by using a Micromeritics Tristar 3000 nitrogen adsorption-desorption analyzer. Transmission electron microscopy (TEM) images were obtained using a JEM-3010 microscope at an accelerating voltage of 300 kV. X-ray photoelectron spectroscopy (XPS) spectra were recorded by using a Thermo Scientific escalab 250xi photon-electron spectrometer. The single-atom features were observed on a JEM-ARM200F spherical aberration-corrected transmission electron microscope platform. Transient photocurrent curves and electrochemical impedance spectra (EIS) were recorded by a ChI660C electrochemical workstation based on a conventional three-electrode system using a 0.1 M Na_2_SO_4_ electrolyte. The Pt electrode was used as the counter electrode, and the Ag/AgCl electrode was used as the reference electrode. Fluoride tin oxide (FTO) conductor glass coated with as-prepared catalysts and Nafion solution was used as the working electrode. The light source for the transient photocurrent and EIS measurements is a 300 W Xe lamp with a 420 nm cutoff filter. A 300 W Xe lamp with 420 nm cutoff filter solid-state ^13^C nuclear magnetic resonance (NMR) spectra was acquired on a Bruker Avance III 400 NMR spectrometer. Electron paramagnetic resonance (EPR) signals were determined on a Bruker EPR A300 spectrometer. The content of Cu in the as-developed catalysts was determined by using an Agilent 7900 inductively coupled plasma spectroscopy-mass spectrometry (ICP-MS) system.

### Photocatalysis performance evaluation of Cu-g-C_3_N_4_ catalysts

The photocatalysis performance of the as-developed Cu-g-C_3_N_4_ catalysts and controls was first determined by the photocatalytic degradation of the RhB dye as a model of refractory organics. A 300 W Xe lamp with a 420 nm cutoff filter (radiation intensity about 50 mW/cm^2^) was used as the simulated visible light source. The reaction was performed in 50 ml of RhB dye aqueous solution (20 mg·L^−1^) filled in a transparent quartz test tube with 10 mg of catalysts. The 30 min dark adsorption was performed before light irradiation to ensure adsorption equilibrium. The solution sample was taken out every 30 min, centrifuged, and measured by using a UV-Vis spectrophotometer. For the measurement of cycle photocatalytic stability, the used photocatalysts were collected by centrifugation and reused another three times.

The photocatalytic H_2_ evolution test was conducted with a hermetic quartz reactor. In a typical procedure, the catalyst (30 mg) was added into a 10% TEOA aqueous solution (100 ml). Before light irradiation, the reactor with the solution was evacuated for 30 min to remove air completely. A simulated solar light (100.00 mW/cm^2^) was provided to maintain the light energy input.

### DFT calculation

Spin-polarized DFT calculations were performed using the CASTEP package with the Perdew-Burke-Ernzerhof generalized gradient approximation exchange-correlation functional. The Cu-CN samples containing 48–50 atoms (including one Cu, 18 C, 27 N, and two O atoms) were used as models. The core electrons were treated with ultrasoft pseudopotentials. Given the calculation cost, geometrical optimization was conducted only at the gamma point.

## Results and discussions

In this work, we hope to obtain a monatomic structure of a defective carbon nitride photocatalyst by low-temperature and high-energy plasma treatment in the atmosphere containing oxalic acid and copper salt. The establishment of this assumption should first ensure the stability of the structure and photoelectric properties of polymer photocatalytic materials and then explore in detail the existence of possible monatomic sites and their binding characteristics so as to provide an experimental basis for the discussion of the mechanism and the potential of new plasma technology.

**FIGURE 2:**
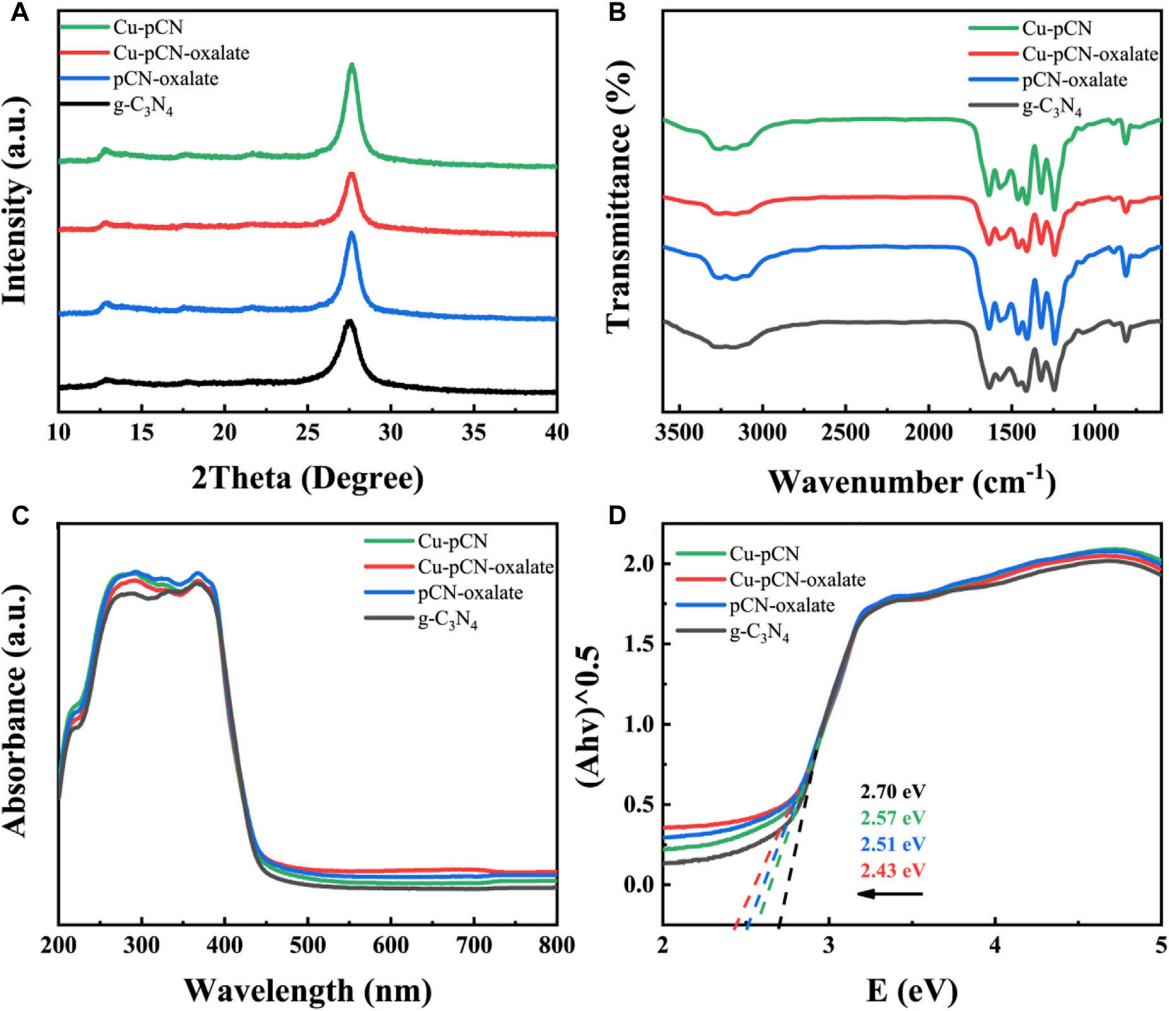
**(A)** XRD patterns, **(B)** FT-IR spectra, and **(C)** UV-Vis spectra of the Cu-modified g-C_3_N_4_ samples by a plasma method. **(D)** Kubelka-Munk transformed band gap diagram of all the samples.

Therefore, we first analyzed the X-ray diffraction (XRD) patterns of the samples before and after plasma treatment. The XRD patterns of the Cu-modified g-C_3_N_4_ samples prepared by the plasma method are consistent with those of the raw g-C_3_N_4_ sample (Figure 2A). Two obvious diffraction peaks can be seen, namely, the (100) peak corresponding to the plane-plane repeating unit at 2θ = 13.1° and the (002) peak relating to the conjugate and aromatic cyclic graphite stacking system at 2θ = 27.6°. The results reflect the graphitic-like layer structure still existing on the plasma-treated g-C_3_N_4_ materials. (Liu N. et al., 2019).

The surface functional groups of all the samples were characterized by FT-IR to verify whether the polymer structure of the Cu-CN-COOH sample (mainly Cu-pCN-oxalate) was altered after the synthetic introduction of Cu species and -COOH group process (Figure 2B). All samples exhibited typical tri-s-triazine peaks at 802 cm^−1^ and between 1250 and 1580 cm^−1^, confirming no alteration to the covalent bonds between carbon and nitrogen during the synthetic process. As previously reported, the slightly emerged peak at 1690 cm^−1^ in the FT-IR spectrum of Cu-pCN-oxalate and pCN-oxalate, as well as the wide peak at around 3000–3300 cm^−1^, indicates the presence of carboxyl groups (Wang S. et al., 2019). The around 3000–3300 cm^−1^ peak in Cu-pCN-oxalate is lower than that of pCN-oxalate, suggesting the -COOH group is evaluated from oxalate as proposed and the -COOH groups have strong interaction with copper in the presence of SAC copper.

The UV-Vis light absorption properties of raw g-C_3_N_4_, Cu-pCN-oxalate, and control samples were investigated, as shown in Figure 2C. Compared with g-C_3_N_4_, the light absorption in the visible light region of Cu-pCN-oxalate was enhanced. The plots of (Ahλ)^1/2^ vs photoenergy (Figure 2D) were obtained through the Kubelka–Munk transformation of UV-Vis spectra. The band gap values (*E*g) of g-C_3_N_4_ and Cu-pCN-oxalate are 2.70 and 2.43 eV, respectively. Obviously, by introducing Cu species and -COOH groups into the raw g-C_3_N_4_ *via* the plasma method, the visible light energy harvesting ability of the Cu-pCN-oxalate photocatalyst is greatly promoted so as to enhance the catalytic performance (Shi et al., 2019). The promoted visible light energy harvesting ability of the Cu-pCN-oxalate photocatalyst can be ascribed to the introduction of monatomic copper and carboxyl defects, which regulate the highest occupied molecular orbital (HOMO) of holes and the lowest unoccupied molecular orbital (LUMO) of electrons of g-C_3_N_4_ polymer semiconductors by changing the charge balance state of N atoms in the two-dimensional plane, leading to a narrowed energy level gap (*E*g) (Dou et al., 2019; Sakuna et al., 2022; Liu Y. et al., 2019). In addition, the enhancement of light absorption near the infrared region is not directly caused by the change of band gap width but can be attributed to the possible Cu-Nx coordination structure.

**FIGURE 3:**
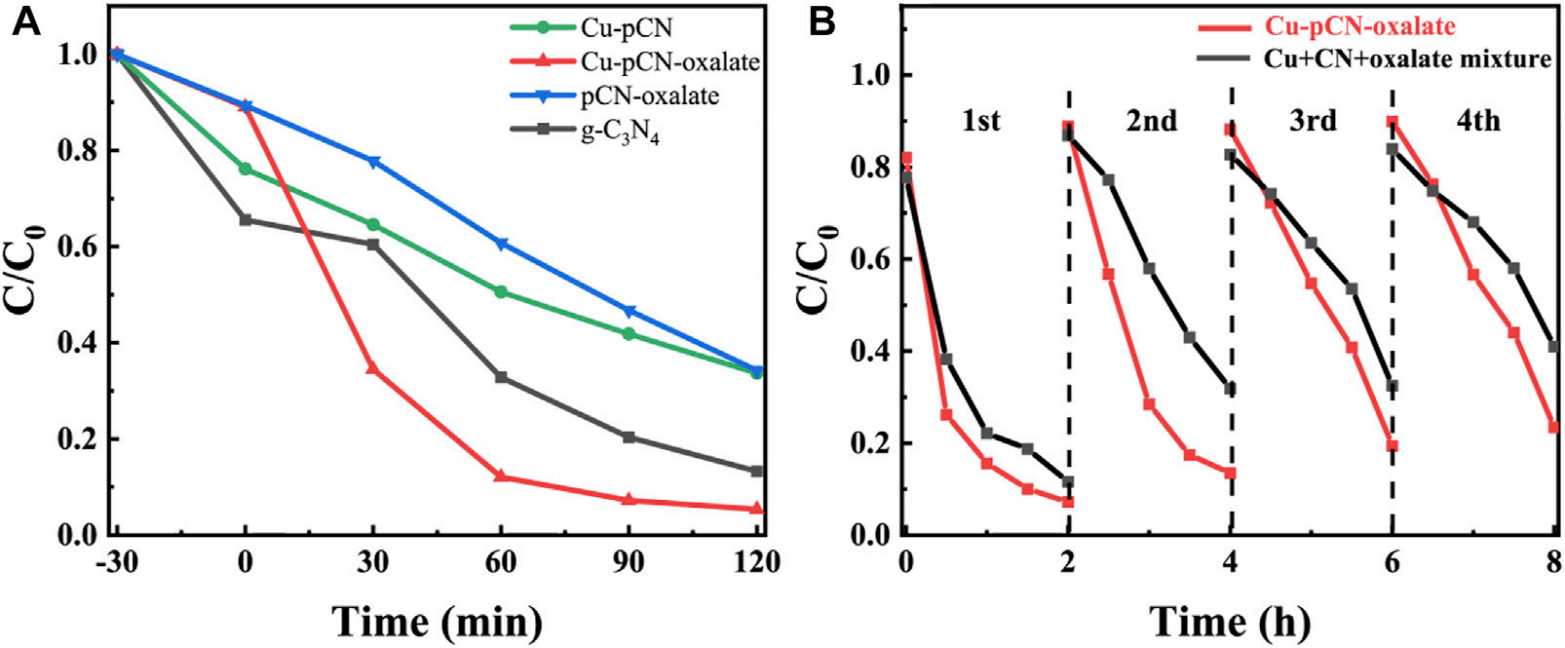
**(A)** Photocatalytic RhB degradation raw g-C_3_N_4_, Cu-pCN-oxalate, and controls. The light was turned on from time 0 min. **(B)** Cycling photocatalytic RhB removal performance of Cu-pCN-oxalate and the mixture control (copper salt, raw g-C_3_N_4,_ and oxalate).

Since the basic physical and chemical properties of the materials have not changed significantly after doping copper and carboxyl precursors with plasma treatment, we directly used dyes to conduct preliminary photocatalytic performance tests in order to expect whether the possible strategy of fixing monatomic catalysts at defect sites can significantly improve the catalytic effect. The carboxyl-deficient photocatalyst loaded with potential monatomic copper (Cu-pCN-oxalate) showed high photocatalytic RhB degradation performance, while the photocatalytic performance of control samples without adding copper or oxalic acid improved slightly (Figure 3A), which proved the necessity of adding copper salt and oxalic acid in the plasma process at the same time. The cycle stability experiment shows that this performance improvement is not caused by simple mixture composition interference but by a continuous catalytic effect (Figure 3B). Specifically, although the mixture may have a certain Fenton effect by using the trace hydrogen peroxide produced by the photocatalysis reaction due to the presence of copper, so the performance of the mixture is better in one cycle, but it fades rapidly in multiple cycles. However, the cycle stability of the Cu-pCN-oxalate sample treated with plasma is significantly improved. Therefore, we believe that the proposed strategy of constructing stable SAC copper photocatalysts based on carboxyl defects has been initially successful. Further morphological characterization, structural analysis, and mechanism study are still needed.

**FIGURE 4:**
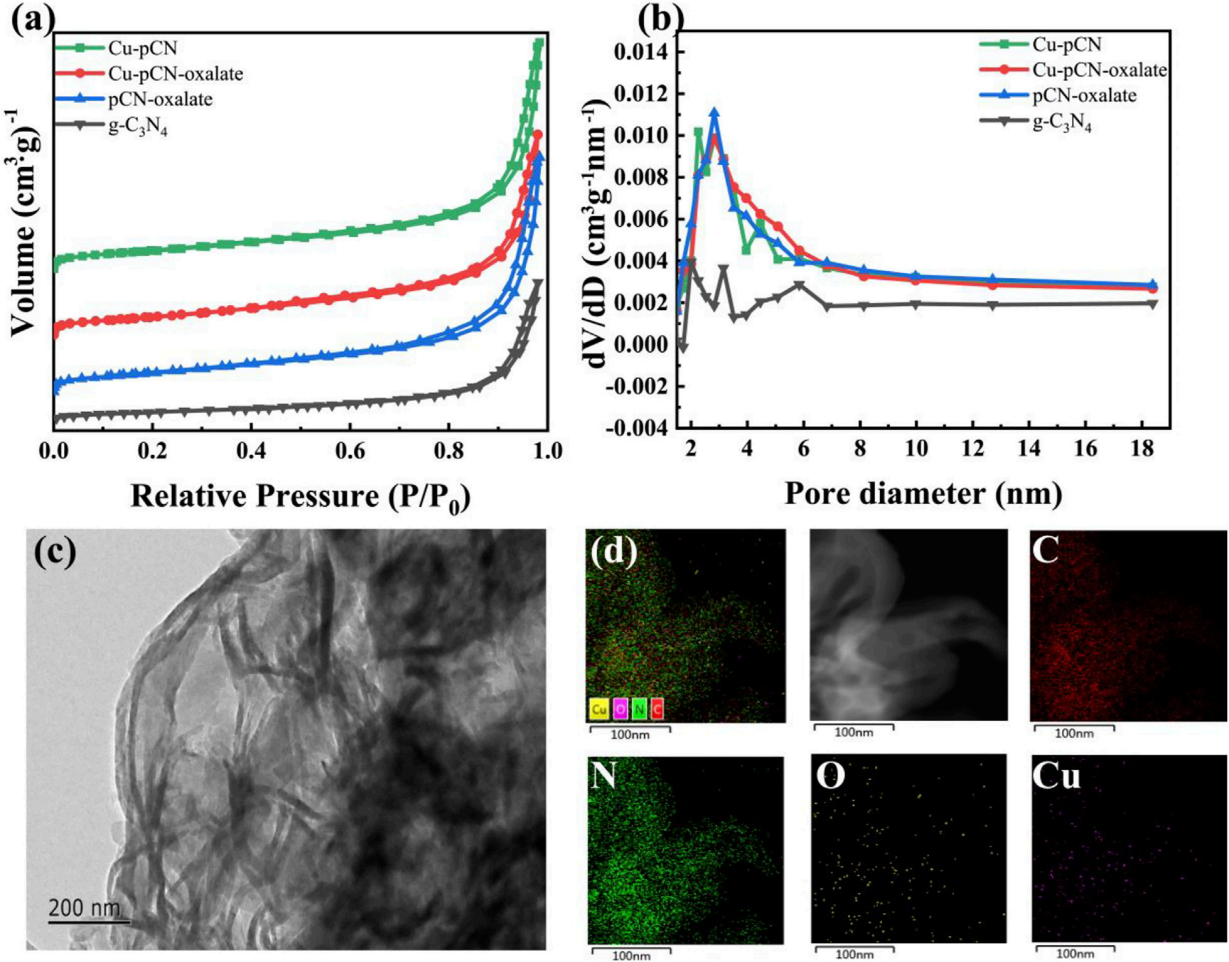
**(A)** Nitrogen adsorption-desorption isotherm and **(B)** corresponding pore size distribution curves of all samples. **(C)** TEM images of the optimal Cu-pCN-oxalate sample with **(D)** corresponding EDS mapping images.

The pore structures of the samples before and after plasma treatment were analyzed by using the nitrogen adsorption-desorption test. The nitrogen adsorption-desorption isotherm is shown in Figure 4A, and the corresponding pore size distribution curves of all samples can be seen in Figure 4B. In general, the adsorption and desorption characteristics of the samples before and after plasma treatment only change slightly. However, it should be noted that the hysteresis loops and pore size distribution of the plasma-treated samples are more obvious, indicating an optimized mesoporous structure. The TEM images of the optimal Cu-pCN-oxalate sample are shown in Figure 4C. Cu-pCN-oxalate exhibited a loose layer stacked mesoporous structure, which could be attributed to the aggregation of carbon nitride nanosheets after the plasma boosting process (Jia et al., 2022). The EDS mapping images of Cu-pCN-oxalate as shown in Figure 4D clearly showed the presence of C, N, O, and Cu. The Cu doping content of the Cu-pCN-oxalate sample is 0.42 wt% according to the ICP measurement. Although the contents of copper and oxygen are low, their distribution is highly uniform, suggesting the possibility of monatomic catalysts.

**FIGURE 5:**
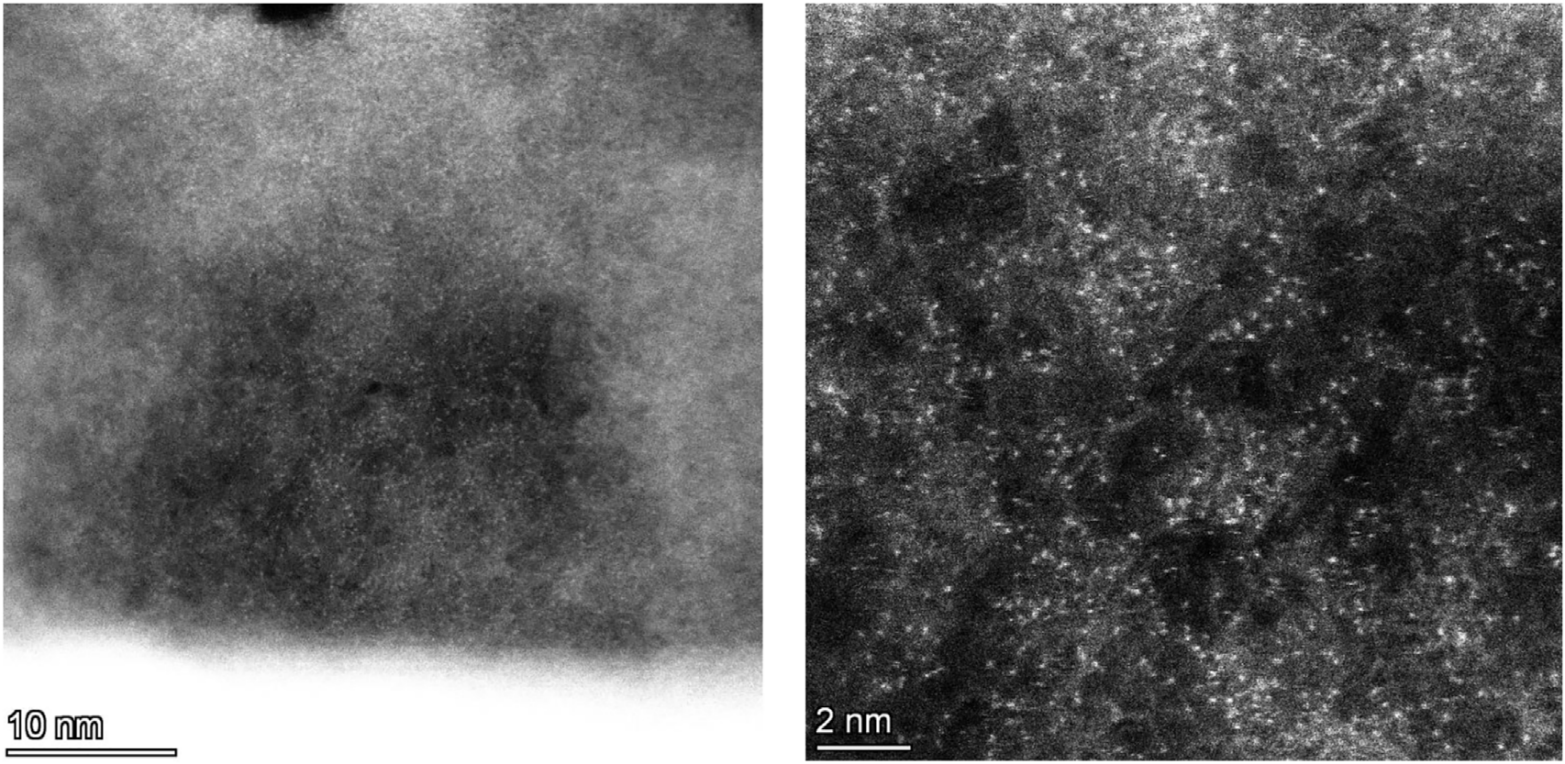
Spherical aberration-corrected TEM images of Cu-pCN-oxalate with strong single-atom features.

In order to directly verify the existence of monatomic copper, we used spherical aberration-corrected transmission electron microscopy to observe the plasma-treated Cu-pCN-oxalate sample, as shown in Figure 5. It is impressive that the samples are rich in monatomic catalyst sites under high-resolution electron microscopy. In the amplification part, the diameter of the bright spot is less than 0.2 nm, which is a very typical morphological feature of monatomic copper (Li et al., 2020; Liu F. et al., 2019). This high-density, ultra-uniform distribution of the monatomic catalyst state is no less than other chemical or physical methods reported in the existing literature (Tong et al., 2018). The most important reason for the successful synthesis of monatomic catalysts may be that the introduction of the carboxyl precursor oxalic acid provides a defective carbon nitride microenvironment suitable for the dispersion of monatomic copper. This study preliminarily explored the proportion of a supported monatomic copper catalyst, but it may have great potential in higher content of the high-quality SAC Cu. We will systematically explore this possibility in our future work.

**FIGURE 6:**
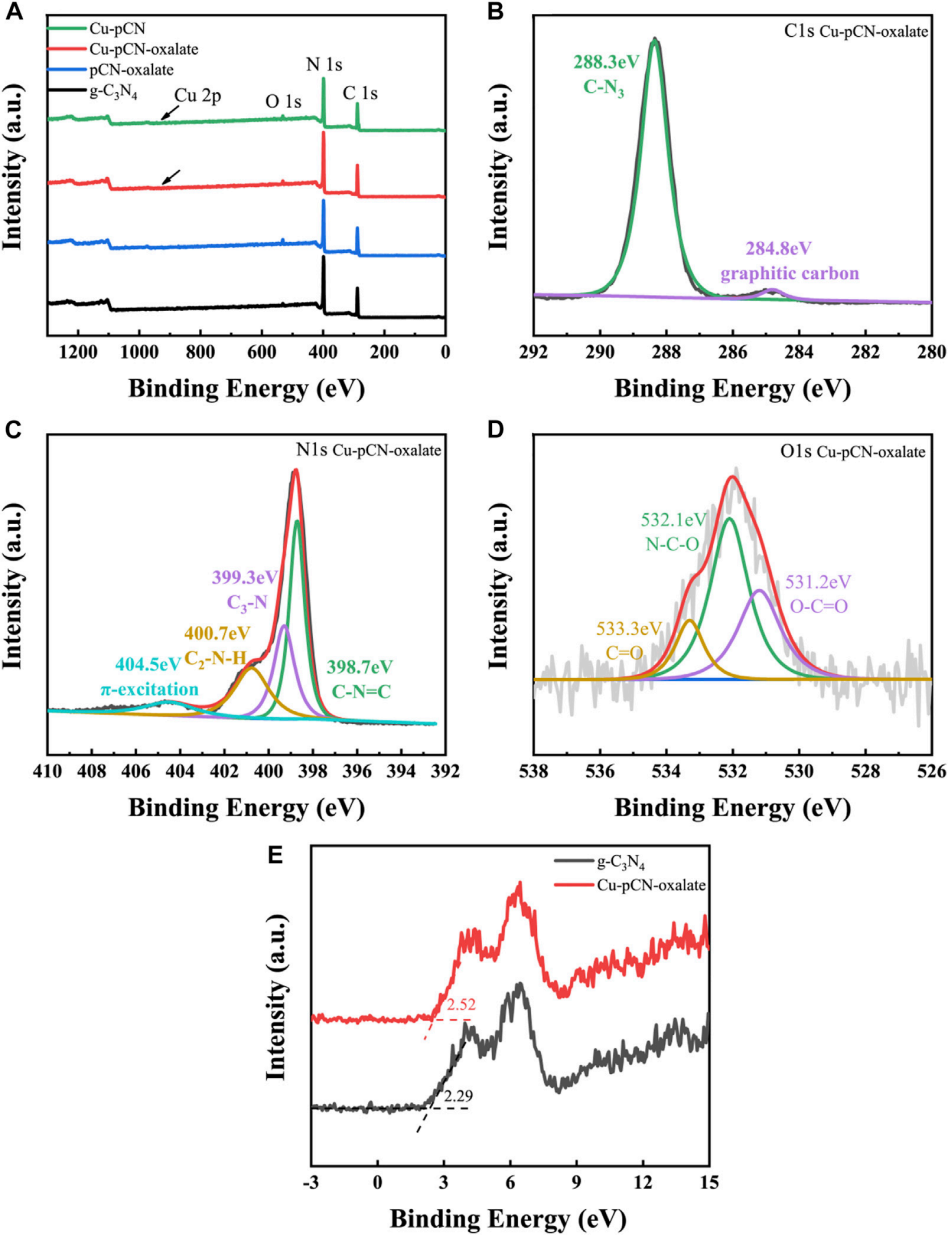
**(A)** XPS survey spectra of raw g-C_3_N_4_ and Cu-pCN-oxalate samples and **(B**–**D)** corresponding high-resolution C 1s, N 1s and O 1s XPS spectra of Cu-pCN-oxalate. **(E)** XPS valence band spectra of raw g-C_3_N_4_ and Cu-pCN-oxalate.

In order to further study the possible existing states of the introduced structures such as monoatomic copper and a carboxyl group, we carried out XPS analysis. Due to the low content of copper (less than 1% mass fraction), the high-resolution fine scanning spectrum of copper cannot be accurately collected. Compared to the literature and our previous work, the XPS survey spectra of the samples prepared by plasma post-treatment are mostly the same as those of the basic samples (Figure 6A). The high-resolution C 1s and N 1s XPS spectra of Cu-pCN-oxalate (Figures 6B,C) also show a conventional feature of raw g-C_3_N_4_. However, there are obvious carboxyl characteristic peaks in high-resolution O 1s spectra of Cu-pCN-oxalate (Figure 6D) compared with those of the conventional g-C_3_N_4_ (Wang D. et al., 2022; Saka, 2022). The presence of the carboxyl group can be further analyzed by ^13^C NMR spectra analysis. The XPS valence band positions (*E*_VB_) of g-C_3_N_4_ and Cu-pCN-oxalate are measured and calculated as 2.29 and 2.52 eV (Figure 6E), respectively. Based on the band gap of the two samples (2.70 eV for g-C_3_N_4_, 2.43 eV for Cu-pCN-oxalate), the band gap structure can be obtained. The HOMO position of Cu-pCN-oxalate becomes more negative, indicating that the excited electrons of Cu-pCN-oxalate have a much stronger reduction ability for photocatalytic H_2_ evolution.

**FIGURE 7:**
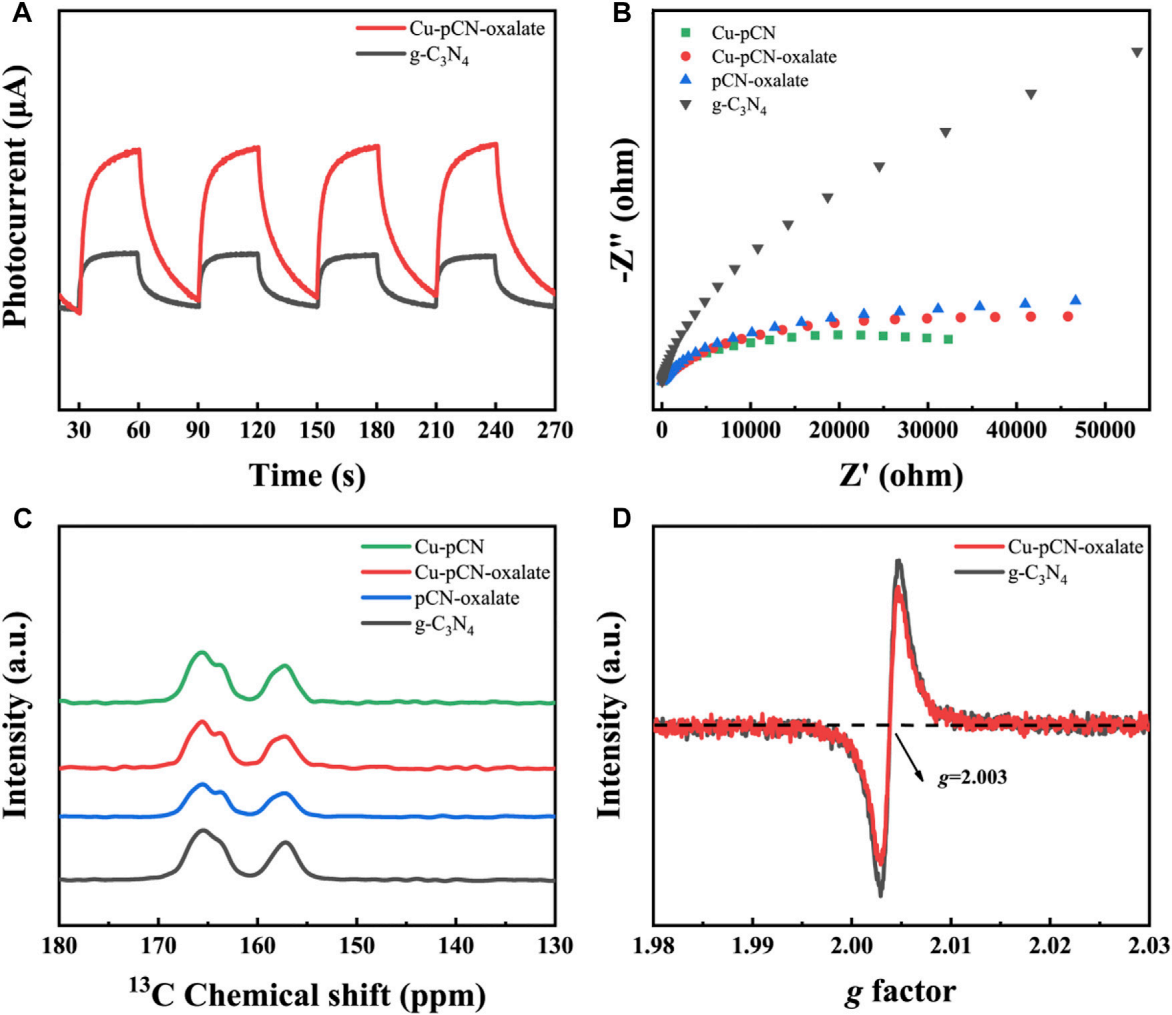
**(A)** Photocurrents and **(B)** EIS spectra of raw g-C_3_N_4_, Cu-pCN-oxalate, and controls. **(C)** Solid-state ^13^C NMR spectra of Cu-pCN-oxalate samples and **(D)** solid-state EPR spectra of raw g-C_3_N_4_ and Cu-pCN-oxalate.

In the promotion of photocatalytic performance, in addition to the expansion of optical absorption, the more important thing is the separation efficiency of photogenerated carriers. This carrier separation efficiency can usually be characterized by measuring the photocurrent and electrochemical impedance spectroscopy of the working electrode in a three-electrode system under illumination. Figure 7A and Figure 7B showed photocurrents and EIS spectra of raw g-C_3_N_4_, Cu-pCN-oxalate, and controls. Obviously, the electrochemical impedance of several materials after plasma treatment is significantly reduced, and the photocurrent of the optimal sample is also significantly stronger than that of the original material, which indicates that the plasma method and material system proposed in this study have excellent performance in improving the photocatalyst’s photogenerated electrons and hole separation (Xiao et al., 2021). The better EIS behavior of the Cu-pCN than the Cu-pCN-oxalate sample can be ascribed to the fact that the high-energy plasma bombardment could format small particles of copper from the copper salt in raw materials, which can reduce the interface resistance. In the Cu-pCN-oxalate sample, a strong reactive metal–support interaction between the SAC Cu and the -COOH defects instead of Cu nanoparticles in the g-C_3_N_4_ may have dominated this promotion, which is more beneficial for photocatalysis applications.

In order to further prove the existence of carboxyl defect structure, which is the structural basis for this excellent carrier separation efficiency, we used solid-state ^13^C NMR analysis. It can be observed that a broad weak peak at around 153 ppm is present in the solid-state ^13^C NMR spectra of Cu-pCN-oxalate (Figure 7C), suggesting the generation of carboxyl groups in the melon units of the g-C_3_N_4_ photocatalyst.

Meanwhile, the environment of the copper element could be judged from the value of the g-tensor parameter (g), in which a g of less than 2.3 can be verified as a covalent state rather than an ionic state (Wu et al., 2015). The measured g value was 2.01, which is less than 2.3, indicating that copper was possible covalently bound onto the main units of the g-C_3_N_4_ photocatalyst.

**FIGURE 8:**
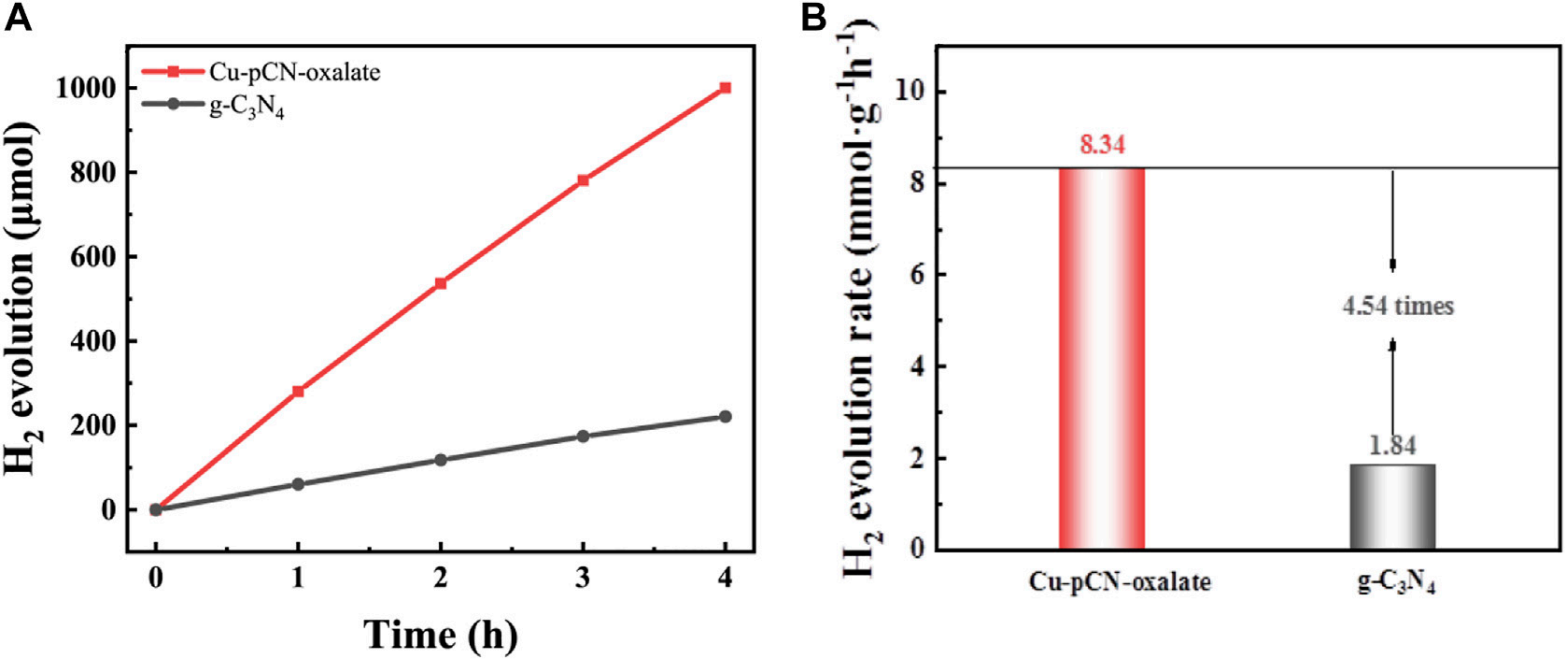
**(A)** Photocatalytic H_2_ production amount and **(B)** average H_2_ evolution rate of raw g-C_3_N_4_ and the optimal Cu-pCN-oxalate catalyst with ideal single-atom Cu and -COOH defects. The reactions were performed in 10 vol% TEOA solution under visible light irradiation (*λ* ≥ 420 nm) (30 mg of the catalyst used in each experiment).

Since it has been suggested in the previous article, due to the change of energy band structure, the obtained catalyst is more suitable for the reduction reaction. At the same time, considering the importance of photocatalytic hydrogen production to solve the energy problem, we carried out photocatalytic hydrogen production experiments with a basic raw g-C_3_N_4_ sample and an optimal Cu-pCN-oxalate sample, as shown in Figure 8. The gas chromatography determined H_2_ evolution rates over the g-C_3_N_4_ samples before and after plasma treatment were 1.84 and 8.34 mmol h^−1^g^−1^, respectively. The photocatalytic H_2_ evolution results reveal the significant photocatalytic performance enhancement of the rapid one-step plasma method in the presence of copper salt and oxalic acid precursor, in which carboxyl defective structure can be generated by the ionization of oxalic acid, thus serving as the immobilization structure and strong reactive metal–support interaction synergetic site to develop high-quality SAC Cu endowed carboxyl-deficient g-C_3_N_4_ photocatalysts.

### Mechanism discussion

Solar-driven photocatalysis plays an important role in solving the energy crisis and environmental pollution. Although the photocatalysis system has been more mature, the catalytic performance is far from meeting the requirements due to the rapid recombination of electron-hole pairs and the small active surface area. Monatomic catalysts provide maximum atomic utilization, enhance light absorption and electron-hole pair separation, and regulate the interaction with intermediates and thus have excellent photocatalytic performance.

Monatomic catalysts combine the advantages of homogeneous catalysis, independent and single active center, 100% atom utilization, stable heterogeneous catalysis, and easy separation and recovery and thus can solve the difficult problems in catalysis science. A strong electronic coupling occurs between the active center and the surrounding atoms in a monatomic catalyst. Studies show the local coordination environment of a single atom critically impacts its electronic structure and catalytic behavior. Therefore, regulating the coordination environment around monatomic active sites is conducive to optimizing the intrinsic activity of monatomic catalysts to meet the needs of directional reactions (Wu et al., 2021).

When the size of metal particles is reduced to the monatomic level, the rapid increase in surface free energy will lead to agglomeration. Therefore, constructing a stable monatomic catalyst is of scientific significance. Research on the preparation of monatomic catalysts has been carried out, and many advances have been made in wet chemistry, metal-organic framework material template method, defect anchoring, atomic deposition, ball milling, and thermal conversion. Recently, changing the coordination environment of monatomic active centers by introducing special defect structures is an effective strategy to regulate intrinsic activity. The existence of special defect structures will affect the electronic structure of monatomic active sites through remote regulation. For example, after introducing carboxyl defects into the g-C_3_N_4_ substrate, different push–pull electron interactions will affect the charge density of monatomic active sites, thus impacting the interaction strength with hydrogen evolution active species and improving the reaction activity. In this case, we believe that the strong reactive metal-support interactions between the SAC Cu and the -COOH defects in the g-C_3_N_4_ promoted the stability and carrier separation of modified photocatalysts (Wang L. et al., 2022; Ojha et al., 2019).

The results of this study confirm that our self-built microwave surface wave plasma device is effective. It is reasonable that the plasma strategy can controllably change the surface characteristics and energy band structure of polymer semiconductors. In terms of the fundamental mechanism of plasma physics, plasma treatment is an interface process of ion injection to modify polymer semiconductors. Ion injection is usually carried out by immersing the material in the plasma, such as the g-C_3_N_4_ with a loose porous structure. After the formation of a sheath layer at the interface between the plasma and material partition, the ions within it are accelerated by the sheath layer, resulting in a strong injection effect that can reach a certain depth, leading to strong and uniform material modification (Conrad et al., 1987; Inagaki et al., 1995).

**FIGURE 9:**
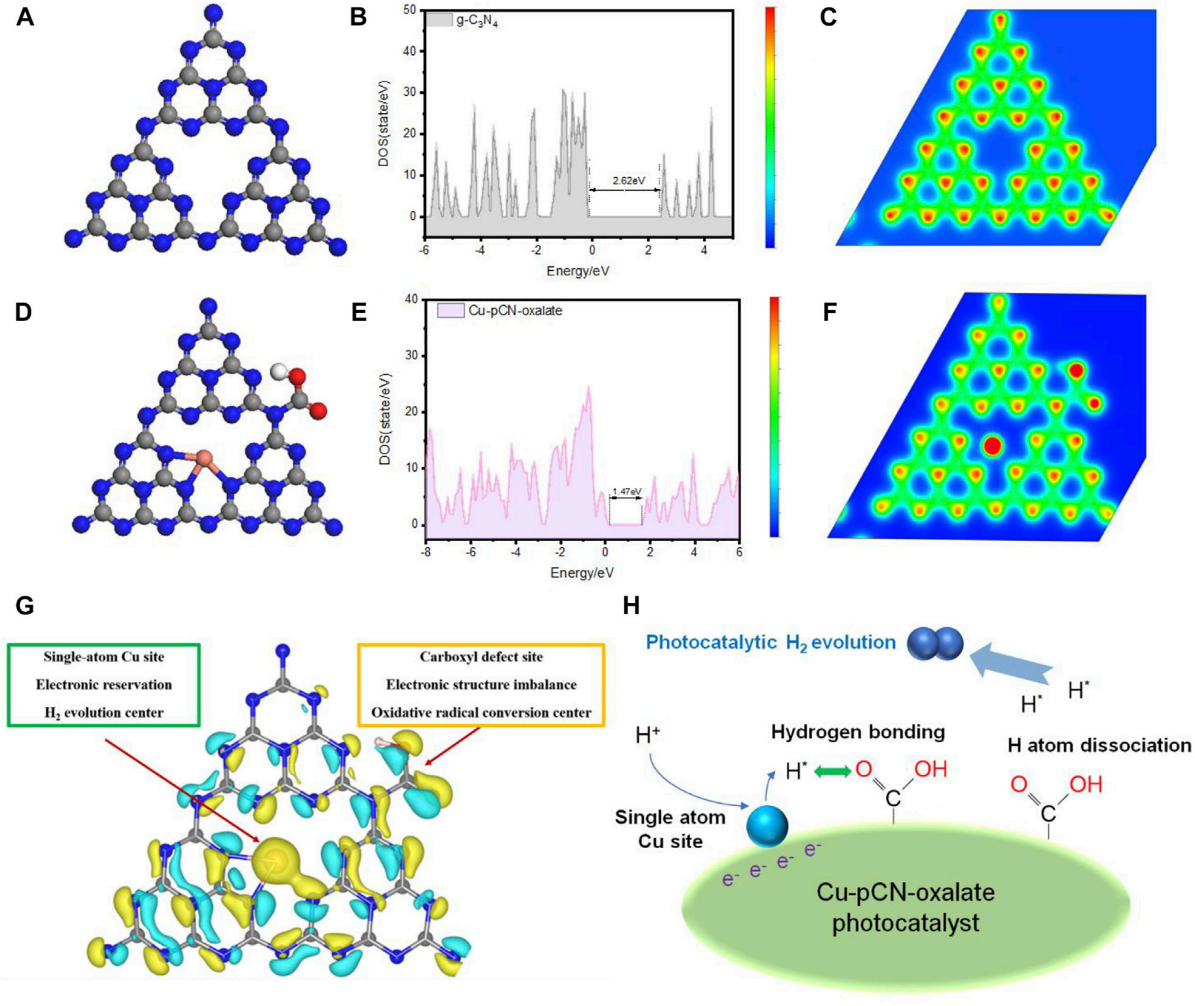
Structure diagrams and corresponding total density of states of **(A**,**B)** raw g-C_3_N_4_ and **(D**,**E)** Cu-pCN-oxalate and the schematic diagram of local charge analysis of **(C)** raw g-C_3_N_4_ and **(F)** Cu-pCN-oxalate (gray, blue, pink, and red spheres: C, N, Cu, and O, respectively). **(G)** Proposed synergistic mechanism of single-atom Cu sites and the carboxyl defect sites of the Cu-pCN-oxalate photocatalysts in a typical 3D map of differential charge densities of Cu-pCN-oxalate. **(H)** Proposed reaction mechanism involving the single-atom Cu sites and the carboxyl defect sites.

Figures 9A–F illustrated the structure diagrams and corresponding total density of states of raw g-C_3_N_4_ and Cu-pCN-oxalate. In terms of the system developed here, we believe through synchrotron radiation, spherical aberration electron microscopy, and DFT calculation that the possibility of a Cu-N_3_ structure is reasonable (Figure 9G). In our opinion, the mechanism of single-atom and defect site combined to improve the effect of the sample compared with the original g-C_3_N_4_ lies in collaborative optimization of electronic structure and the optimization of interface transmission. In terms of collaborative electronic structure, the single-atom Cu sites with strong electronic reservation could serve as hydrogen production centers, and the carboxyl defect sites with complex imbalance local electronic structure could be the oxidative radical conversion field, which can provide a separate derivative site for various free radicals. The coexistence of two unbalanced electronic structures (monatomic copper and carboxyl defect) led to different intensities and electron-rich electrophilic binding sites for various photocatalytic reactions and resulted in a boosted charge separation ability (Sun et al., 2022). In terms of interface transmission mechanism, a possible reaction pathway of H_2_ evolution was proposed and illustrated in Figure 9H: 1) the electron-rich monatomic copper site reduces H^+^ to H atom; 2) the H atom is transferred to the carboxyl site (because it easily forms hydrogen bonds); 3) the H atom on the carboxyl group is unstable and can produce H_2_ (Liang et al., 2020).

In this process, the unique carboxyl structure plays an important role. The carboxyl group contains a carbonyl group and a hydroxyl group. The atom connected with the carboxyl group is coplanar with one carbon atom and two oxygen atoms in the functional group, and the hydrogen atom is out-of-plane. Both oxygen atoms in the carboxyl group have lone pair electrons, which can attract hydrogen atoms to form hydrogen bonds. The aforementioned steps constitute a key H atom transfer process, avoiding the accumulation of H atoms at the single-atom copper active site and the photogenerated carrier recombination caused by the slow reaction of photoelectrons. In a word, the oxalic acid promoter-assisted plasma method proposed here provides a new possible route for ultrafast and large-scale universal preparation of carboxyl-deficient transition-metal monatomic catalysts. This new technology and strategy has broad application prospects in the fields of energy and catalysis.

## Conclusion

A rapid plasma method for synthesis of single-atom Cu-supported defective g-C_3_N_4_ by using oxalic acid as a carboxyl precursor (Cu-pCN-oxalate) is reported. This material shows high reactivity and stability in photocatalytic Fenton degradation of pollutants and photocatalytic hydrogen evolution. Spherical aberration electron microscopy clearly shows the new material has very high density and highly dispersed monatomic copper sites. The mechanism of photocatalytic hydrogen evolution of Cu-pCN-oxalate was proposed by X-ray absorption spectroscopy, electrochemistry characterizations, and DFT calculations. The strong reactive metal-support interactions between the transition-metal atoms and the -COOH defects in the g-C_3_N_4_ host not only ensured the stability of the composite system but also supported the local electronic structure reconstruction, thus leading to a boosted charge separation ability. The synergy interface transmission mechanism involving the single-atom Cu sites and the carboxyl defect sites of the Cu-pCN-oxalate photocatalysts during the H_2_ evolution process was also proposed. This work provides a deep understanding of the design of monatomic photocatalysts and offers a production strategy with industrial prospects.

## Data Availability

The raw data supporting the conclusions of this article will be made available by the authors, without undue reservation.
